# Exogenous Production of Silver Nanoparticles by *Tephrosia apollinea* Living Plants under Drought Stress and Their Antimicrobial Activities

**DOI:** 10.3390/nano9121716

**Published:** 2019-12-01

**Authors:** Muna A. Ali, Kareem A. Mosa, Ali El-Keblawy, Hussain Alawadhi

**Affiliations:** 1 Department of Applied Biology, College of Sciences, University of Sharjah, Sharjah P.O. Box 27272, UAE; u17105693@sharjah.ac.ae (M.A.A.); akeblawy@sharjah.ac.ae (A.E.-K.); 2 Department of Biotechnology, Faculty of Agriculture, Al-Azhar University, Cairo 11651, Egypt; 3 Center for Advanced Materials Research, Research Institute of Sciences and Engineering, University of Sharjah, Sharjah P.O. Box 27272, UAE; halawadhi@sharjah.ac.ae

**Keywords:** silver nanoparticles, living plants, green synthesis, drought stress, phytosynthesis, antimicrobial activity

## Abstract

Nanoparticle (NP) synthesis by biological systems is more cost-effective, safe, and environmentally friendly when compared to currently used chemical and physical methods. Although many studies have utilized different plant extracts to synthesize NPs, few studies have incorporated living plants. In this study, silver nanoparticles (AgNPs) were synthesized exogenously by *Tephrosia apollinea* living plant system under the combined stresses of silver nitrate and different levels of drought stress simulated by Polyethylene glycol (PEG) (0, −0.1, −0.2, and −0.4 MPa for three and six days). Biomass, cell death, and H_2_O_2_ content were evaluated to determine the toxicological effect of the treatments on the plant. More severe effects were detected in day 6 plants compared to day 3 plants, and at higher drought levels. UV-visible spectrum, energy dispersive X-ray spectroscopy, X-ray diffraction, scanning electron microscope, and Fourier transform infrared spectroscopy were used to detect and characterize the *T. apollinea* synthesized NPs. The shapes of the NPs were spherical and cubic with different phytochemicals being the possible capping agents. Broth microdilution was used to determine the antimicrobial activity of the NPs against *Escherichia* *coli* and *Staphylococcus aureus*. In this case, antimicrobial activity increased at higher PEG concentrations. Bactericidal effects were observed against *E. coli*, while only bacteriostatic effects were detected against *S. aureus.*

## 1. Introduction

Nanoparticles (NPs) are used in various applications, including environmental, medical, nanobiosensors, agriculture, and clinical clothing [1]. Among the various types of NPs, silver nanoparticles (AgNPs) are one of the most commercialized, comprising over 50% of global nanomaterial-based market products available in 2015 [2]. AgNPs are used widely due to their unique antimicrobial, optical, electromagnetic, and physicochemical properties [3]. The synthesis of NPs is dependent upon chemical- or physical-based methods that are expensive and require harmful chemicals despite their efficiency [4]. In addition, these methodologies require harsh environments for the synthesis reaction to occur, including extremes of temperature, pressure, and pH [5].

Recently, biological methods were utilized as a green method for NP synthesis, involving the use of biological systems like bacteria, fungi, yeast, algae, and plants [6]. These methods are considered more cost-effective, safe, and eco-friendly [7]. In addition, they require ambient conditions like room temperature, atmospheric pressure, and physiological pH [8]. Among biological systems, living plants are renewable, safe to handle and easily available resources for nanoparticle production [1]. In contrast to bacteria, plant systems eliminate the need to frequently maintain cell cultures [8]. Extracts of different plant species have been utilized to synthesize NPs as they comprise different biomolecules that act as reducing agents [6]. AgNPs synthesized by plant extracts have a variety of applications in the medical field. Examples of plant extracts utilized to synthesize AgNPs with antimicrobial activity include *Cressa cretica* [9] and *Azadirachta indica* [10]. Anticancer activity was found in AgNPs synthesized using the extracts of *Cyperus conglomeratus* [11] and *Syzygium aromaticum* [12]. In addition, extracts of *Tephrosia tinctoria* [13] and *Argyreia nervosa* [14] demonstrated promising antidiabetic activities.

Although plant extracts have been widely used for nanoparticle synthesis [15], only a few studies have investigated the synthesis of AgNPs using living plants. In comparison to the production of NPs using plant extracts, living plants can reduce the spillover of toxic chemicals into the environment [3]. Thus, living plants are promising resources for nanoparticle synthesis that can meet the growing market demand for nanoparticle production. The synthesis of NPs depends on reducing agents and phytochemicals which play an important role in the reduction of Ag^+^ to Ag^0^ and the subsequent stabilization of the formed NPs [7]. Different plant species release different groups of phytochemicals. It has been reported that *Vigna radiata* plants treated with AgNO_3_ exhibited reduced levels of proteins and sugars, suggesting their probable role in NP synthesis [3].

*Tephrosia apollinea* (Delile) DC. is a perennial shrub native to the United Arab Emirates that grows in the lower mountain regions [16]. It has been used as a traditional medicine to relieve nasal congestion, earache, wounds, and bone fractures [17]. In addition, the plant has demonstrated insecticidal, anticancer and antibacterial properties [18]. In this regard, antibacterial compounds like tephrosin, deguelin, and quercetin were isolated from different *Tephrosia* spp. [17]. Furthermore, extracts of *Tephrosia* spp. were used for the synthesis of metal NPs. The stem extract of *Tephrosia tinctoria* was also utilized to synthesize AgNPs with promising antidiabetic properties [13]. In addition, AgNPs were synthesized from the leaf extract of *Tephrosia purpurea* and found to possess antimicrobial properties [19]. Antibacterial gold NPs were also synthesized using the leaf extract of *T. purpurea* [20].

In this study, the ability of *T. apollinea* to exogenously synthesize AgNPs is reported under the combined stress of silver ions and different levels of drought based on plant treatments. Furthermore, the antimicrobial activity of the isolated AgNPs was investigated. To the best of the authors’ knowledge, this is the first report to investigate the antimicrobial activities of NPs synthesized by living plant roots, as well as the first time that living *T. apollinea* plants were employed for the biosynthesis of NPs.

## 2. Materials and Methods

### 2.1. Seed Germination

Seeds of *T. apollinea* collected from Wadi Al-Ejili, Ras Al-Khaima in the United Arab Emirates were immersed in concentrated sulfuric acid for 25 min with stirring. Subsequently, they were washed several times with running tap water and then with distilled water. Seeds were then soaked in distilled water and incubated in a growth chamber adjusted at 30 °C/20 °C (12 h day/12 h night) at medium light intensity for 24 h. Accordingly, the seeds were germinated on filter papers in petri plates with 5 mL of distilled water and incubated until radical emergence. Seedlings were then transferred to strainers in 100 mL beakers filled with distilled water. Five plants were grown in each beaker. They were incubated at 30 °C/20 °C (12 h day/12 h night) at medium light intensity. After one week, the distilled water was replaced with 15% Hoagland’s solution with a final pH of 5.7–5.8. During incubation, Hoagland’s solution was added as needed. After seven weeks, plants were treated with solutions as explained in the subsequent section.

### 2.2. Preparation of Treatment Solutions and Plant Harvest

*T. apollinea* plants were treated with 15% Hoagland’s media with 1 mM AgNO_3_ alone or with different PEG concentrations corresponding to −0.1 MPa, −0.2 MPa, and −0.4 MPa. The control solutions contained only 15% Hoagland’s media. These PEG levels were selected based on a preliminary experiment tested the tolerance of *T. apollinea* to osmotic stress. The final pH of all the solutions was adjusted to 5.7–5.8. Eight replicas were prepared for each treatment, and the plants were incubated in the same conditions described above. The day of adding the treatment solutions was considered as day 0. Four plant replicas of each treatment were harvested on day 3, and the other four replicas were harvested on day 6. Plants were harvested by separating the shoots and roots, and the fresh biomass was measured. The plant tissues were stored at −80 °C until being ground in liquid nitrogen to form a fine powder which was stored at −80 °C for further analysis. The electrical conductivity (EC) of the growth media solutions were measured at day 0, day 3, and day 6 respectively. After plant harvest, the solutions were kept in the dark at room temperature.

### 2.3. Evan’s Blue Staining

The control and treated plant root tips were stained with 0.25% (*w*/*v*) of Evan’s blue (Himedia) in 0.1 M CaCl_2_ for 15 min with gentle shaking at room temperature. Subsequently, roots were washed three times with CaCl_2_ followed by three further washings with distilled water and observed using a bright-field microscope (Optika B-1000 BF, Ponteranica, Italy).

### 2.4. Measurement of H_2_O_2_ Content

H_2_O_2_ content was measured as described previously [21] with slight modifications. 100 mg of frozen roots were homogenized with 1 mL of 0.1% trichloroacetic acid (TCA) solution and then centrifuged at 13,000× *g* for 10 min at 4 °C. The clear supernatant (50 µL) was mixed with 50 µL of 10 mM potassium phosphate buffer (pH 7) and 100 µL of 1 M potassium iodide, with the absorbance measured at 390 nm (Epoch™ 2 Microplate Spectrophotometer, BioTek Instruments, Inc., Winooski, VT, USA). The H_2_O_2_ content was determined using an extinction coefficient of 0.28 µM^−1^ cm^−1^.

### 2.5. Nanoparticle Characterization

The solutions obtained from the replicas of each treatment were combined and centrifuged at 5500 RPM for 20 min; the supernatant was discarded, and the pellet was washed once with deionized water and air-dried. The pellets were analyzed by XRD (D8 Advance, Bruker AXS, Karlsruhe, Germany) with step size 0.03°, time/step = 0.50 s, and Cu kα as the X-ray source. In addition, the pellets were analyzed by FT-IR (FT/IR 6300, Jasco, Tokyo, Japan). 1 mg of the pellet was dispersed in 500 µL deionized water and characterized with UV-vis spectrophotometry (Epoch™ 2 Microplate Spectrophotometer, BioTek Instruments, Inc., Winooski, VT, USA), with the wavelength range between 300 nm and 700 nm. The prepared nanoparticle suspensions were sonicated for 30 min (XUBA Analogue Ultrasonic Bath, Grant instruments, Cambridgeshire, United Kingdom). 10 µL of the sonicated suspension was placed on the aluminum slide and left to air dry. SEM and EDS analyses were carried out using a TESCAN VEGA3 XM SEM, SE Detector, 30 kV, high vacuum (TESCAN, Brno – Kohoutovice, Czech Republic).

### 2.6. Antimicrobial Broth Microdilution Assay

The synthesized AgNPs were tested for their antimicrobial activities against *E. coli* (ATCC 25922) and *S. aureus* (ATCC 29213). The minimum inhibitory concentration (MIC) was determined using a broth microdilution assay prepared according to the Clinical and Laboratory Standards Institute (CLSI) [22,23]. Briefly, 100 µL of 2000 µg/mL nanoparticle suspension was added to the first column of a 96-well plate and 1:2 dilution series with Mueller Hinton Broth (MHB) was performed. Then, overnight cultures of *E. coli* and *S. aureus* grown on nutrient agar at 37 °C were diluted with sterile normal saline to OD600 of 0.1, resulting in a suspension of 1–2 × 10^8^ colony forming units (CFUs) mL^−1^. Subsequently, the bacterial suspension was diluted by 1:10 and 5 µL of the diluted suspension was added to each well. In each plate, a row containing only MHB and a column containing bacterial suspension in the absence of NPs served as negative and positive controls respectively. Each treatment was tested in triplicates. The plate was incubated in a shaker at 37 °C overnight and bacterial growth was observed by visual inspection. The MIC was considered as the lowest concentration of NPs that inhibited the visible growth of bacteria. To determine the minimum bactericidal concentration (MBC), an aliquot of 3 µL obtained from all wells showing no visible bacterial growth was spotted onto nutrient agar plates and incubated overnight at 37 °C. MBC was defined as the lowest concentration that completely inhibited bacterial growth on the plate [24]. The broth microdilution experiment was repeated three times and the obtained MIC and MBC values were representative of the typical results.

### 2.7. Statistical Analysis

The student’s *t*-test was used to assess the significant difference between the mean values obtained from treated and control plants respectively. A *p*-value < 0.05 was considered as statistically significant. The student’s *t*-test was done using R [25]. The obtained data were categorized based on the *p*-value and indicated with *: *p* ≤ 0.05, and **: *p* ≤ 0.01. R packages of ggplot2 [26] and dplyr [27] were used to create certain graphs. Error bars in the figures represent the standard error.

## 3. Results and Discussion

### 3.1. Effects of AgNO_3_ and PEG on Plant Phenotype and Biomass

**Figure 1:**
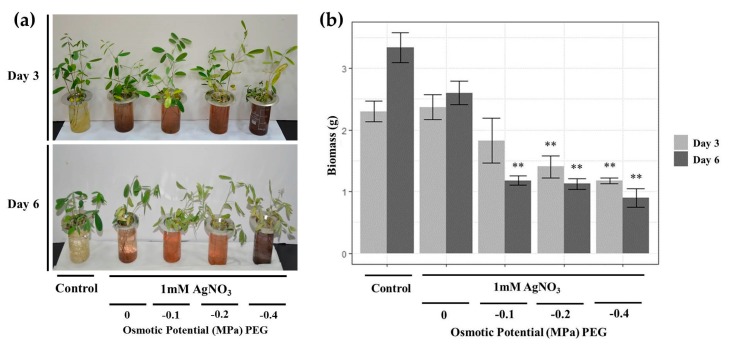
The effect of AgNO_3_ and Polyethylene glycol (PEG) treatments on (**a**) plant phenotypes and (**b**) biomass of *T. apollinea* at day 3 and day 6 of treatment with 1 mM AgNO_3_ alone or with different concentrations of PEG corresponding to −0.1, −0.2, and −0.4 MPa.

Control plants featured green healthy leaves on both days 3 and 6. The phenotype of the plant leaves treated with 1 mM AgNO_3_ were similar to control plants on day 3 but demonstrated chlorosis and started to wilt by day 6. Plants treated with 1 mM AgNO_3_ and PEG wilted and demonstrated yellowish leaves, with the severity of symptoms dependent on increased PEG concentrations as well as increased incubation times Figure 1a. On day 0, all the solution colors were transparent. However, on day 3 and day 6, the solution colors of treated plants turned brown, with darker colors observed in plants treated with PEG of −0.4 MPa. The alteration of media color from transparent to brown or dark brown indicates the formation of AgNPs [5,28]. The variation in solution colors might be due to differences in sizes and shapes of the formed NPs [29].

Fresh biomass was measured to evaluate the effect of AgNO_3_ and PEG treatments on *T. apollinea* growth Figure 1b. *T. apollinea* plants treated with 1 mM AgNO_3_ showed similar biomass as control plants on both day 3 and day 6. On the other hand, plants treated with different concentrations of PEG had reduced biomass in comparison to control plants. Increased plant biomass reduction was observed to be in accordance with increased PEG concentration. Biomass reduction under drought stress is commonly observed among plants [30]. Additionally, *Cassia angustifolia* plants treated with different concentrations of PEG also revealed reduced biomass, with more severe symptoms demonstrated at higher PEG concentrations [31]. A similar result was obtained in investigations where *Sesuvium portulacastrum* was treated with PEG [32]. Treatment with AgNO_3_ also led to a significant reduction in the biomass of *Spirodela polyrhiza* [33] and *Brassica* sp. [34] respectively.

### 3.2. AgNO_3_ and PEG Exhibited Increase in T. apollinea EC

**Figure 2:**
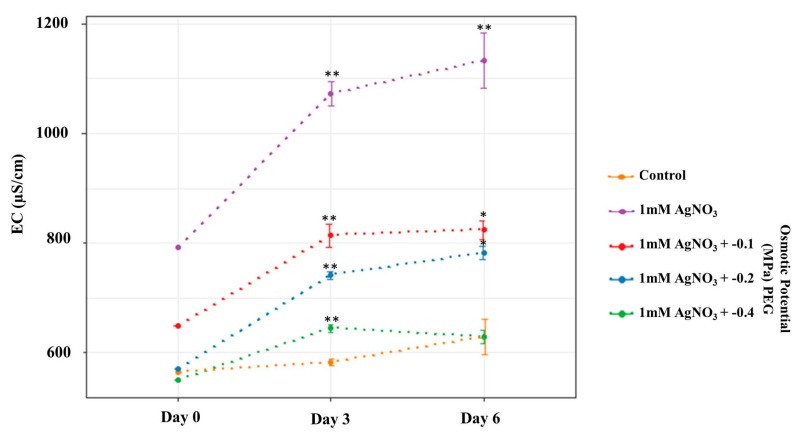
Electrical conductivity of the control and the treated plant media. The figure represents the EC values of the prepared solution on day 0, and the average electrical conductivity (EC) on days 3 and 6 respectively. This figure was created using R [25].

For all treated plants, the EC of the media increased on days 3 and 6 respectively compared to day 0, as shown in Figure 2. This increase in EC may be attributed to the attractive interactions between AgNPs and silver ions, suggesting increased AgNP numbers in the media [35,36]. EC was also observed to reduce under increasing PEG concentrations. This may be due to the presence of more unreacted silver ions at lower PEG concentrations [37]. In addition, on days 0, 3, and 6, the EC of control plant solutions was lower in comparison to treated plants, except at 0.4 PEG on day 6 which was similar to the control EC value. The lower EC values at higher PEG concentrations may be related to the interactions of the ions with PEG [38].

### 3.3. AgNO_3_ and PEG Stresses Reduced Viability of T. apollinea Root Cells

**Figure 3:**
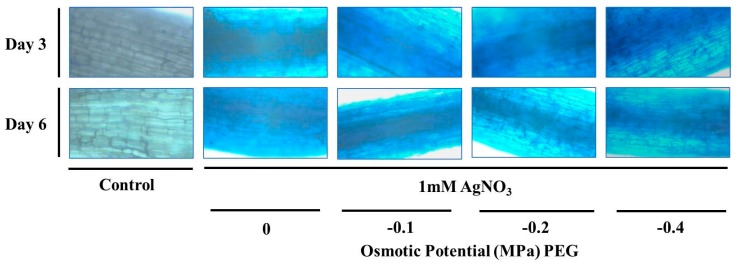
Evan’s blue staining assay of *T. apollinea* roots treated with 1 mM AgNO_3_ alone or with different concentrations of PEG corresponding to −0.1, −0.2, and −0.4 MPa respectively.

Root tips were stained with Evan’s blue to detect cell membrane damage, which reflects cell death. In this case, viable cells exclude while dead cells retain Evan’s blue stain. Plants treated with either AgNO_3_ alone or different concentrations of PEG showed increased uptake of Evan’s blue stain (indicated by the intense blue color obtained) demonstrating reduced cell viability Figure 3. Cell death is caused by different types of abiotic stresses, including drought and metals. As such, exposure of *Sorghum bicolor* to drought stress resulted in reduced cell viability [39]. Furthermore, the treatment of *Pandanus odorifer* with silver ions or AgNPs caused root cell death [40]. Similarly, *Coriandrum sativum* plants treated with different concentrations of copper NPs also revealed root cell death [41].

### 3.4. AgNO_3_ and PEG Exposure Caused Oxidative Stress in T. apollinea Roots

**Figure 4:**
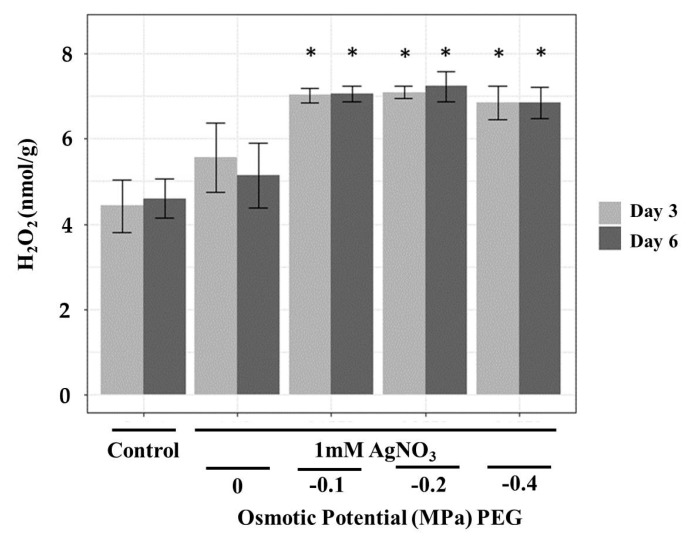
H_2_O_2_ contents in the roots of *T. apollinea* plants treated with 1 mM AgNO_3_ alone or with different concentrations of PEG corresponding to −0.1, −0.2, and −0.4 MPa. This figure was created using R [25].

H_2_O_2_ is one of the reactive oxygen species (ROS) produced under drought stress [42]. It causes many deleterious effects to plants including lipid peroxidation, disruption of cellular metabolic function as well as affecting cellular integrity [43]. H_2_O_2_ levels in *T. apollinea* roots were increased significantly in treated plants Figure 4. This indicates that the treatments caused oxidative stress in *T. apollinea* roots. A similar pattern was observed in *Coriandrum sativum* plants treated with copper NPs, where only the roots were oxidatively stressed [41]. Exposure of *Cucumis sativus* plants to copper NPs also leads to a significant H_2_O_2_ increase in roots [44].

### 3.5. Detection of Exogenous AgNPs Synthesised by T. apollinea Plants

**Figure 5:**
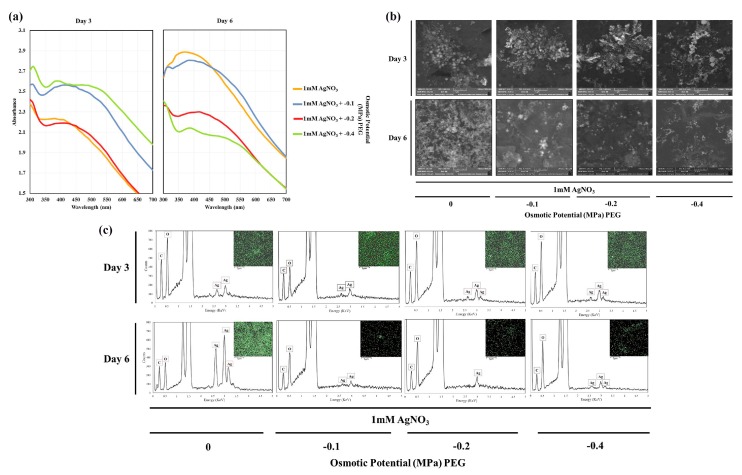
Detection of phytosynthesized AgNPs by *T. apollinea* using (**a**) Uv-vis, (**b**) SEM, and (**c**) EDS. Plants were treated with 1 mM AgNO_3_ alone or with different concentrations of PEG corresponding to −0.1, −0.2, and −0.4 MPa.

The formation of exogenous AgNPs was detected by the Uv-vis absorption spectra of treatment suspensions. Broad surface plasmon resonance (SPR) peaks between 370–430 nm were observed on both day 3 and day 6, as shown in Figure 5a. The SPR spectra depends on the size, morphology, and composition of the NPs [45]. According to previous studies, absorption at this range is due to the SPR of AgNPs [45,46]. In addition, the position of the absorption peak varies depending on plant species [28].

SEM results showed the presence of NPs in the prepared colloidal solutions of treated plants on both days 3 and 6 Figure 5b. Most of the formed NPs were round, with some cubic NPs also observed. EDS and element mapping results indicated the presence of silver in the NPs Figure 5c. The EDS peaks around 3 KeV indicates the presence of silver [47,48]. In addition, the resulting peaks of carbon and oxygen were possibly due to organic phytochemicals that acted as capping agents of the synthesized NPs. This was observed in silver nanoparticles synthesized by the extract of *Kappaphycus alverazii* [49] and the cell-free filtrate of *Aspergillus flavus* NJP08 [50]. The additional peak of aluminium was due to the aluminium slide used in EDS.

### 3.6. XRD Analysis of the Synthesized Nanoparticles

**Figure 6:**
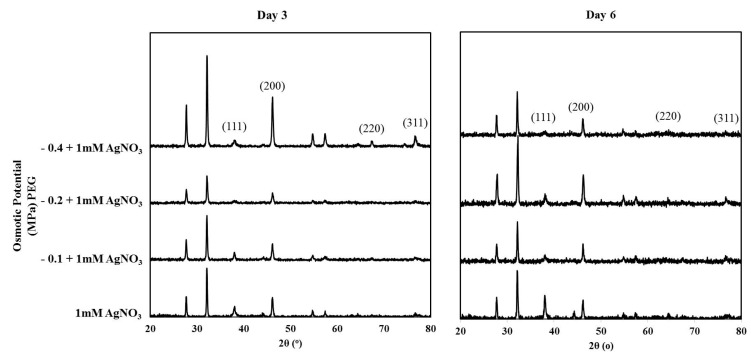
XRD patterns of the AgNPs synthesized exogenously by *T. apollinea* at day 3 and day 6. Plants were treated with 1 mM AgNO_3_ alone or with different concentrations of PEG corresponding to −0.1 MPa, −0.2 MPa, and −0.4 MPa. The XRD patterns showed diffraction peaks corresponding to the planes of Ag^0^ and Ag_2_O based on JCPDSV cards of Ag^0^ and Ag_2_O.

The crystalline nature of the synthesized NPs was confirmed using XRD Figure 6. In all treatments, peaks were found at 2θ values of 38°, 46°, 67°, and 76°, which correspond to the (111), (200), (220), and (311) planes of the face-centered cubic (fcc) silver crystal, respectively (JCPDS no. 00-04-0783) [51]. In addition, there are other peaks that may correspond to silver oxide nanoparticles (JCPDS no. 00-076-1393) [52], which could be resulted from the oxidative leaching of Ag^+^ from already formed AgNPs. This was observed in the green synthesis of AgNPs by 16 different species of living plant systems [28]. Moreover, it was found in AgNPs developed from purple acid phosphatase apoenzyme isolated from *Limonia acidissima* [53].

### 3.7. Encapsulation of AgNPs and T. apollinea Phytochemicals

**Figure 7:**
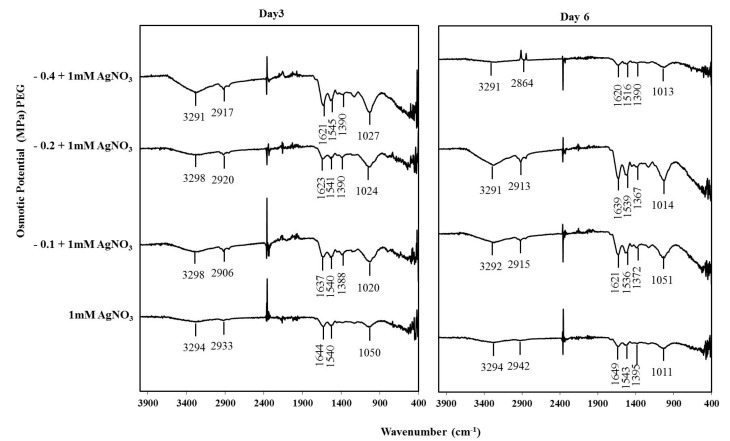
FT-IR spectra of silver nanoparticles synthesized exogenously by *T. apollinea* on day 3 and day 6 respectively. Plants were treated with 1 mM AgNO_3_ alone or with different concentrations of PEG corresponding to −0.1, −0.2, and −0.4 MPa.

FT-IR analysis was performed to identify possible interactions between synthesized AgNPs and plant bioactive molecules that may act as capping and stabilizing agents, as shown in Figure 7. The band around 3300 cm^−1^ could be due to N–H and O–H stretching [53]. The band around 1630 cm^−1^ might indicate C=O of amide I bond, and the bands at 1385 and 1030 cm^−1^ could match with C–N stretching vibrations of the aromatic and aliphatic amines, respectively [54]. The results indicate the binding of proteins serving as capping agents with the synthesized silver nanoparticles. A similar result was obtained previously upon the synthesis of extracellular AgNPs by *Aspergillus flavus* NJP08 [50]. The band around 2920 cm^−1^ could be attributed to O–H stretch of carboxylic acids, which was detected in AgNPs produced by the extract of *Tephrosia tinctoria* plant [13].

### 3.8. Phytosenthesized AgNP Produced by T. apollinea Exhibited Antimicrobial Activities

The synthesized NPs demonstrated antimicrobial activities against both *E. coli* and *S. aureus*. The NPs with the highest antimicrobial activity were synthesized at PEG treatment corresponding to −0.2 MPa day 3 and −0.4 MPa day 3 and day 6, with MIC values of 31.25 μg/mL against *E. coli* and 15.63 μg/mL against *S. aureus* Table 1. This might be a result of more NPs and less unreacted Ag^+^ ions present at higher PEG concentrations, which was concluded based on the EC results. The control plants’ media solution demonstrated no antimicrobial activity against both *E. coli* and *S. aureus*.

The antimicrobial activity of AgNPs was tested widely against several pathogenic bacteria. It has been established that AgNPs possess strong antimicrobial activity against both Gram-positive and Gram-negative bacteria [55]. However, previous investigations have not provided a definite answer on whether Gram-negative [56] or Gram-positive bacteria [57,58] are more sensitive to AgNPs. While most of the *T. apollinea* synthesized NPs in our study were bactericidal against *E. coli*, they were only bacteriostatic against *S. aureus*. The higher sensitivity of Gram-negative *E. coli* to AgNPs compared to gram-positive *S. aureus* is due to the structural features of the bacterial cell wall. The cell wall of Gram-positive bacteria (30 nm) is thicker than Gram-negative bacteria (5–6 nm) due to the presence of multiple layers of peptidoglycan in the cell wall of Gram-Positive bacteria [56]. As a result, gram-negative bacteria may indeed be more susceptible to AgNPs [24].

## 4. Conclusions

AgNPs were successfully synthesized exogenously by *T. apollinea* plants under the combined stress of silver ions and drought on days 3 and 6 respectively. More severe effects on plants were observed at higher PEG levels in relation to biomass and root cell death. Treatments also caused oxidative damage in roots. Most of the characterization results were similar in both NPs isolated after days 3 or 6. The shapes of the NPs were spherical and cubic with different phytochemicals being the possible capping agents involved. The EC values were decreased at higher PEG concentrations, indicating the presence of lower ionic silver. This last factor may explain the reason behind the higher antimicrobial activity of NPs synthesized at PEG treatment corresponding to −0.4 MPa against *E. coli* and *S. aureus* respectively. To the best of the authors’ knowledge, the findings of this manuscript presented for the first time the antimicrobial activity of AgNPs synthesized via living plants. Further studies are required to investigate the other possible applications of such living plant-derived NPs. In addition, more plant stress parameters should be evaluated along with the ability of plants to recover following treatment. It is our hope that such investigations will eventually lead to the possibility of utilizing plants as bio-factories for the sustainable synthesis of NPs.

## Figures and Tables

**Table 1:** The antimicrobial effect of phytosynthesized AgNPs produced by *T. apollinea* plants treated with 1 mM AgNO_3_ alone or with different concentrations of PEG corresponding to −0.1, −0.2, and −0.4 MPa. The values of MIC and MBC (μg/mL) are presented for different types of nanoparticles and silver nitrate against *E. coli* and *S. aureus* respectively.

Treatment			Day	*E. coli*		*S. aureus*	
				MIC	MBC	MIC	MBC
1 mM AgNO_3_			3	125	1000	31.25	>1000
			6	125	500	62.5	>1000
1 mM AgNO_3_+	−0.1	Osmotic Potential (MPa) PEG	3	62.5	250	31.25	>1000
			6	125	250	62.5	>1000
	−0.2		3	31.25	125	15.625	>1000
			6	62.5	500	62.5	>1000
	−0.4		3	31.25	125	15.625	>1000
			6	31.25	125	15.625	>1000
